# Meta-analysis of gene expression in relapsed childhood B-acute lymphoblastic leukemia

**DOI:** 10.1186/s12885-017-3103-1

**Published:** 2017-02-10

**Authors:** Yock-Ping Chow, Hamidah Alias, Rahman Jamal

**Affiliations:** 1 UKM Medical Molecular Biology Institute (UMBI), Universiti Kebangsaan Malaysia Medical Center, 56000 Cheras, Kuala Lumpur Malaysia; 2 Department of Pediatric, Faculty of Medicine, National University of Malaysia, Universiti Kebangsaan Malaysia Medical Center, 56000 Cheras, Kuala Lumpur Malaysia

**Keywords:** Pediatric B-acute lymphoblastic leukemia, Relapse, Microarray, Gene expression

## Abstract

**Background:**

Relapsed pediatric B-acute lymphoblastic leukemia (B-ALL) remains as the leading cause of cancer death among children. Other than stem cell transplantation and intensified chemotherapy, no other improved treatment strategies have been approved clinically. Gene expression profiling represents a powerful approach to identify potential biomarkers and new therapeutic targets for various diseases including leukemias. However, inadequate sample size in many individual experiments has failed to provide adequate study power to yield translatable findings. With the hope of getting new insights into the biological mechanisms underpinning relapsed ALL and identifying more promising biomarkers or therapeutic targets, we conducted a meta-analysis of gene expression studies involving ALL from 3 separate studies.

**Method:**

By using the keywords “acute lymphoblastic leukemia”, and “microarray”, a total of 280 and 275 microarray datasets were found listed in Gene Expression Omnibus database GEO and ArrayExpress database respectively. Further manual inspection found that only three studies (GSE18497, GSE28460, GSE3910) were focused on gene expression profiling of paired diagnosis-relapsed pediatric B-ALL. These three datasets which comprised of a total of 108 matched diagnosis-relapsed pediatric B-ALL samples were then included for this meta-analysis using RankProd approach.

**Results:**

Our analysis identified a total of 1795 upregulated probes which corresponded to 1527 genes (pfp < 0.01; FC > 1), and 1493 downregulated probes which corresponded to 1214 genes (pfp < 0.01; FC < 1) respectively. *S100A8* appeared as the top most overexpressed gene (pfp < 0.01, FC = 1.8) and is a potential target for further validation. Based on gene ontology biological process annotation, the upregulated genes were most enriched in cell cycle processes (enrichment score = 15.3), whilst the downregulated genes were clustered in transcription regulation (enrichment score = 12.6). Elevated expression of cell cycle regulators (e.g kinesins, *AURKA*, *CDK*s) was the key genetic defect implicated in relapsed ALL, and serve as attractive targets for therapeutic intervention.

**Conclusion:**

We identified *S100A8* as the most overexpressed gene, and the cell cycle pathway as the most promising biomarker and therapeutic target for relapsed childhood B-ALL. The validity of the results warrants further investigation.

**Electronic supplementary material:**

The online version of this article (doi:10.1186/s12885-017-3103-1) contains supplementary material, which is available to authorized users.

## Background

B-Acute lymphoblastic leukemia (ALL) accounts for 80% of childhood leukemias, and relapsed B-ALL remains as the leading cause of cancer related deaths among children [1, 2]. Despite the 5-year survival rate for pediatric ALL exceeding 90% after treatment with multi-agent chemotherapy tailored to established risk factors [3], nearly 20% of patients will still relapse and succumb to disease. Relapsed B-ALL has a dismal prognosis, with overall survival rates of 35–40% even when treated with intensified chemotherapy or stem cell transplantation [4–6]. To date, the biological mechanisms of relapsed ALL remains largely unknown. Therefore, there is a pressing need to gain better understanding of the molecular mechanisms governing relapsed ALL, with the hope of developing more effective treatment plans and to improve patients’ survival rate.

In the past decades, microarray has been widely used to identify candidate biomarkers and therapeutic targets by studying the gene expression changes at the genome wide level. Several studies on diagnosis-to-relapsed ALL have been performed to unlock the dysregulated genes and pathways essential in driving relapsed ALL [7–10]. However, only a very small number of genes were found significantly differentially expressed between diagnosis and relapse, and the results were not consistent across all these studies. These discordant results therefore have limited the reliability for further validation or development into clinically useful biomarkers and therapeutic targets. It has been well recognized that small sample sizes, different microarray platforms, and different statistical methods are among the limiting factors contributed to the discordant results. To resolve this limitation, meta-analysis represent a powerful approach to combine different datasets from different studies to improve the reliability and generalizability of the findings by increasing its statistical power analysis. Meta-analysis on gene expression data has yielded new biological insights, as well as identification of more robust and reliable candidate biomarkers and therapeutic targets [11–13].

To identify differentially expressed genes across multiple datasets, we employed a non-parametric ‘rank product’ method [14, 15]. RankProd is among the most popular tool which utilizes a non-parametric statistical method and outperforms other meta-analysis methods, including metaArray [16], GeneMeta [17], and MAMA [18], by ranking the differentially expressed genes based on false discovery rate. Matched diagnosis and relapse samples represent the most ideal biological samples to study the mechanisms for relapse. Hence, in this study, we sought to identify differentially expressed genes associated with relapsed ALL by performing a meta-analysis on three independent microarray datasets of paired diagnosis-relapsed B-ALL, with the hope of providing new insights into the molecular mechanisms of relapsed B-ALL, as well as to identify potential therapeutic options to improve patients’ outcome. Interestingly, our analysis found a long list of significantly differentially expressed genes which have been missed in individual studies, and highlighted cell cycle regulators as promising therapeutic targets amenable for relapsed childhood B-ALL.

## Methods

### Selection of microarray datasets

To identify paired diagnosis-relapsed pediatric B-ALL microarray expression datasets for meta-analysis, we performed a web-based search in Gene Expression Omnibus database GEO (http://www.ncbi.nlm.nih.gov/geo) and ArrayExpress (http://www.ebi.ac.uk/arrayexpress) database using the keywords “acute lymphoblastic leukemia”, and “microarray”. A total of 280 and 275 expression by array datasets were listed in GEO and ArrayExpress databases respectively (before 6^th^ March 2015). The datasets were reviewed manually and only datasets which fulfilled the following criteria were included for further analysis: (1) Expression profiling by array, (2) Studies which comprised of CEL raw files, and (3) Paired diagnosis-relapsed pediatric B-ALL samples. Only 3 microarray datasets were found, in which GSE28460 and GSE18497 were listed in GEO, whilst GSE28460, GSE18497, and GSE3910 were recorded in ArrayExpress. All three microarray datasets were included in this meta-analysis. GSE3910 consisted of 32 matched diagnosis-relapsed ALL using the using the Affymetrix Human Genome U133A Array [8], whilst GSE18497 [9] and GSE28460 [7] were generated using Affymetrix Human Genome U133 Plus 2.0 Array platform, and consisted of 27 and 49 matched diagnosis-relapsed ALL samples respectively.

### Individual microarray data analysis

To identify differentially expressed genes in each individual dataset, the limma package which employs a linear modeling approach was used. The raw CEL files was normalized using Robust Multichip Averaging (RMA) implemented in the Affy package, returning log2 transformed intensities [19]. The normalized datasets were then subjected to limma to compute differentially expressed genes. Genes significantly dysregulated in relapsed ALL as compared to matched data at diagnosis were defined by a *p*-value < 0.05, and log2 fold change of >1 (upregulated genes) or < -1 (downregulated genes). The results of the linear modelling on each dataset and meta-analysis using RankProd method were then compared.

### Meta-analysis of multiple microarray datasets

Meta-analysis was performed on the three datasets using the RankProd package [14] to identify the upregulated and downregulated genes between relapsed ALL and matched samples at diagnosis. Initially, the raw CEL files were normalized using RMA implemented in the Affy package, returning log2 transformed intensities [19]. The normalized datasets were then merged using inSilicoMerging package, and the batch effects was adjusted using method COMBAT [20]. To identify top differentially expressed probesets, the RPadvance function within the RankProd package was used [14]. False discovery rates (pfp) of differential expression were determined using 1000 permutations. The list of upregulated or downregulated probes was identified based on false discovery rate (pfp <0.01) and fold change value (FC > 1, upregulated; FC < 1, downregulated). Probes that mapped to multiple genes were discarded to avoid misinterpretation of the results and to increase the specificity.

### Gene enrichment analysis

Significantly upregulated (FC > 1, pfp < 0.01) and downregulated genes (FC < 1, pfp < 0.01) identified by RankProd were subjected for gene enrichment analysis using the Database for Annotation, Visualization, and Integrated Discovery (http://david.abcc.ncifcrf.gov/) [21] to identify over-represented functional classes of genes. STRING [22] was used to identify the protein-protein interaction network on selected clustered genes.

## Results

### Individual microarray data analysis of differentially expressed probes

**Fig. 1:**
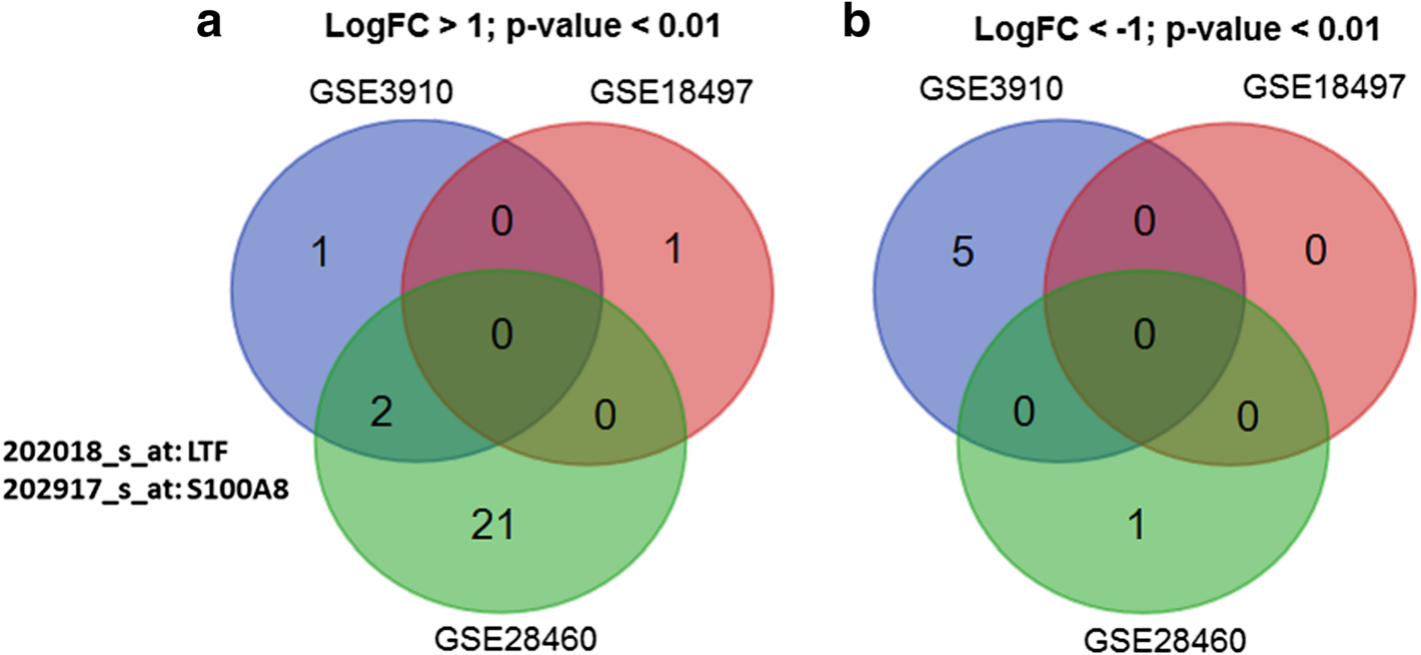
Venn diagram of differentially expressed probes identified from each individual microarray dataset using limma approach. **a** Upregulated probes (*p*-value < 0.01, logFC > 1); **b** Downregulated probes (*p*-value < 0.01, logFC < -1). Only 2 probes which encode for *LTF* and *S100A8* were found concordantly upregulated in 2/3 studies

Differentially expressed genes were identified between relapsed and diagnosed ALL in each study using the limma method which employed the t-test statistical algorithm, and the overlapped genes were examined. As depicted in Fig. 1, based on the cutoff *p*-value <0.05 and logFC > 1, we identified 3 probes which were upregulated in GSE3910, 1 probe in GSE18497, and 23 probes in GSE28460. Of these probes, only 2 probes, i.e. 202018_s_at which encodes for *LTF*, and 202917_s_at which encodes for *S100A8* were found consistently upregulated in 2/3 datasets. In the downregulation profile (*p*-value <0.05 and logFC < -1), no overlapped candidate probe was found. There were 5 probes uniquely downregulated in GSE3910, whereas 1 probe was downregulated in GSE28460 whereas no probe was found significantly downregulated in GSE18497. The genes’ list was as summarized in Additional file 1: Table S1.

### Meta-analysis of differentially expressed probes

To overcome the limitation of small sample sizes in individual study, we then performed meta-analysis on these 3 datasets using RankProd approach. A total of 108 matched diagnosis-relapse ALL samples were pooled together to identify differentially expressed genes implicated in relapsed ALL. The significance of differential gene-expression was calculated based on percentage of false positive predictions (pfp). After removal of probes that mapped to multiple genes or unannotated genes, based on 1000 permutations and a cut-off of false discovery rate at < 0.01, of the 27,000 probes examined, 1795 probes (corresponding to 1527 genes) were found to be upregulated in relapsed ALL (FC > 1), whilst 1493 probes (corresponding to 1214 genes) were downregulated (FC < 1). The top 20 ranked upregulated and downregulated probes are as listed in Tables 1 and 2 respectively, whilst the list of dysregulated probes are as summarized in Additional file 1: Table S2.

**Table 1:** The top 20 most significantly upregulated probes identified by RankProd in relapsed childhood ALL (pfp < 0.01; FC > 1), 1000 permutation

Probe	Gene	FC:(class1/class2)	pfp	*p*.value
202917_s_at	*S100A8*	1.8713	0	0
203949_at	*MPO*	2.0051	0	0
201427_s_at	*SEPP1*	1.6536	0	0
213975_s_at	*LYZ*	1.6129	0	0
205000_at	*DDX3Y*	1.1483	0	0
204971_at	*CSTA*	1.757	0	0
209160_at	*AKR1C3*	1.6693	0	0
209170_s_at	*GPM6B*	1.3387	0	0
204409_s_at	*EIF1AY*	1.2661	0	0
201291_s_at	*TOP2A*	1.8054	0	0
204304_s_at	*PROM1*	1.1579	0	0
201669_s_at	*MARCKS*	1.4776	0	0
202018_s_at	*LTF*	1.6698	0	0
211657_at	*CEACAM6*	1.3632	0	0
212077_at	*CALD1*	1.5944	0	0
221731_x_at	*VCAN*	1.3855	0	0
214039_s_at	*LAPTM4B*	1.1776	0	0
204620_s_at	*VCAN*	1.434	0	0
200665_s_at	*SPARC*	1.4726	0	0
209687_at	*CXCL12*	1.7029	0	0

*FC* fold change, class 1 represent relapsed ALL, class 2 diagnosed ALL

**Table 2:** The top 20 most significantly downregulated probes identified by RankProd in relapsed childhood ALL (pfp < 0.01; FC < 1), 1000 permutation

Probe	Gene	FC:(class1/class2)	pfp	*p*.value
209480_at	*HLA-DQB1*	0.8503	0	0
219737_s_at	*PCDH9*	0.5924	0	0
210432_s_at	*SCN3A*	0.6362	0	0
203038_at	*PTPRK*	0.8517	0	0
206637_at	*P2RY14*	0.7417	0	0
204897_at	*PTGER4*	0.6231	0	0
221728_x_at	*XIST*	0.9313	0	0
212592_at	*IGJ*	0.7981	0	0
221841_s_at	*KLF4*	0.6573	0	0
203910_at	*ARHGAP29*	0.8594	0	0
206864_s_at	*HRK*	0.6284	0	0
210448_s_at	*P2RX5*	0.6183	0	0
214218_s_at	*XIST*	0.9554	0	0
201005_at	*CD9*	0.7092	0	0
210517_s_at	*AKAP12*	0.8163	0	0
205081_at	*CRIP1*	0.605	0	0
204439_at	*IFI44L*	0.7068	0	0
205289_at	*BMP2*	0.7267	0	0

*FC* fold change, class 1 represent relapsed ALL, class 2 diagnosed ALL

Interestingly, in agreement with the linear modeling approach that identified the upregulation of *S100A8* in relapsed ALL (2/3 microarray datasets, Fig. 1), the meta-analysis also detected this candidate probe as the most significantly upregulated target (Table 1). Therefore, *S100A8* appeared to be an attractive and promising biomarker and therapeutic target for relapsed B-ALL that warrants further validation.

**Fig. 2:**
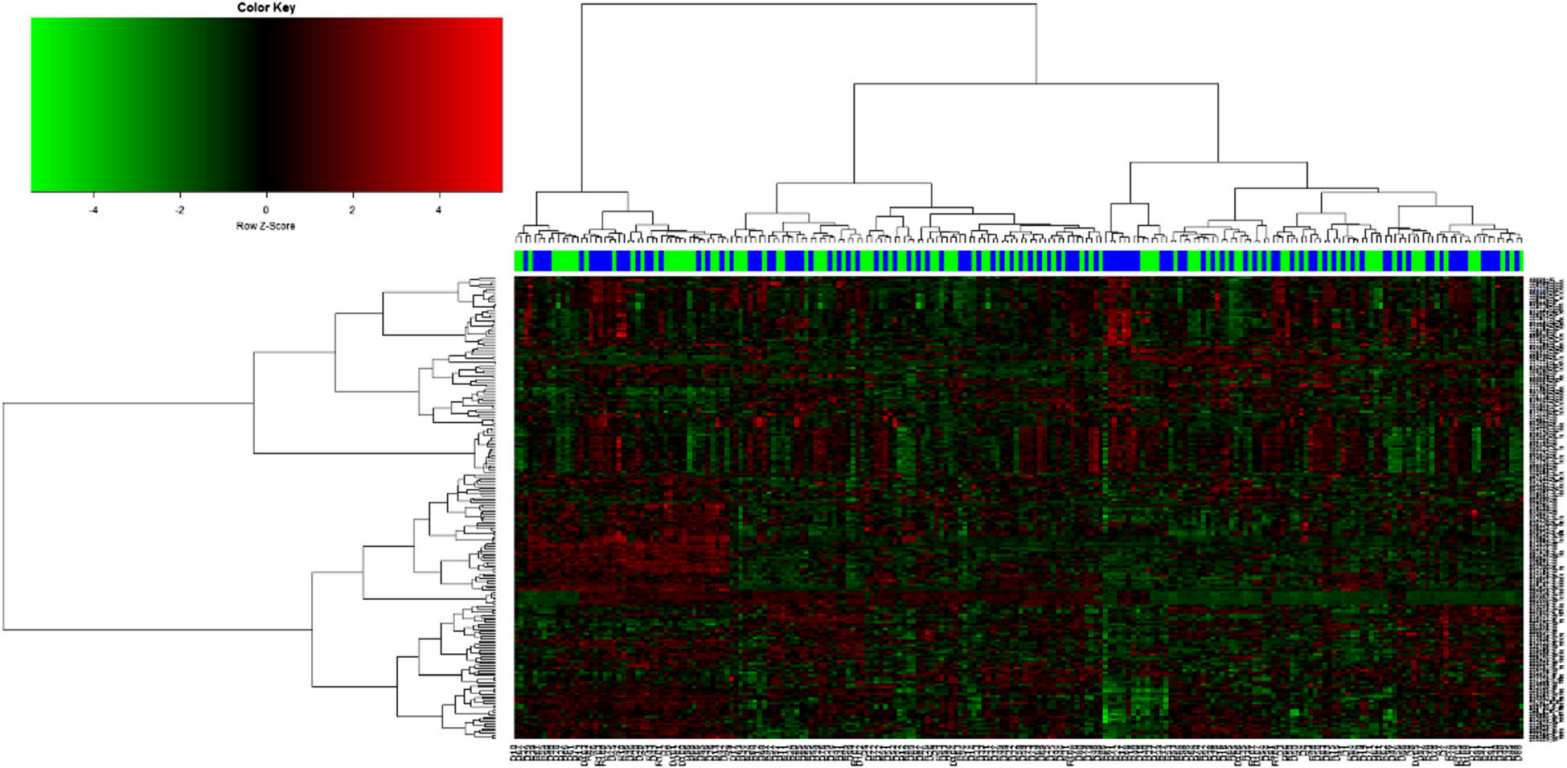
Heatmap of the top 100 differentially expressed probes between relapsed and matched diagnosed B-ALL samples (*n* = 108) from meta-analysis of three microarray datasets. Each green color column denotes newly diagnosed B-ALL samples whilst each blue color column denotes relapse B-ALL samples. Expression levels are represented by red (high expression) and green (low expression)

As shown in Fig. 2, hierarchical clustering on top 100 dysregulated probes of relapsed and diagnosed childhood B-ALL demonstrated that both groups are not clustered uniquely and were mixed together. This profile indicated that the expression profiles of these 2 samples groups were highly similar.

### Functional and pathway analysis

**Fig. 3:**
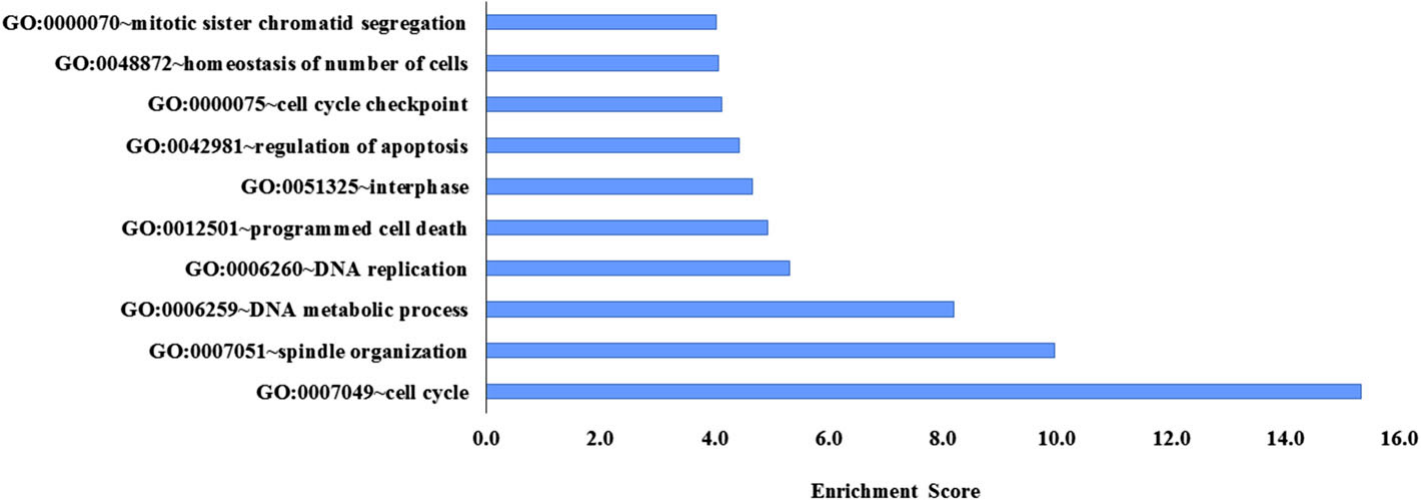
The ten most significant biological processes associated with genes upregulated in relapsed childhood B-ALL

**Fig. 4:**
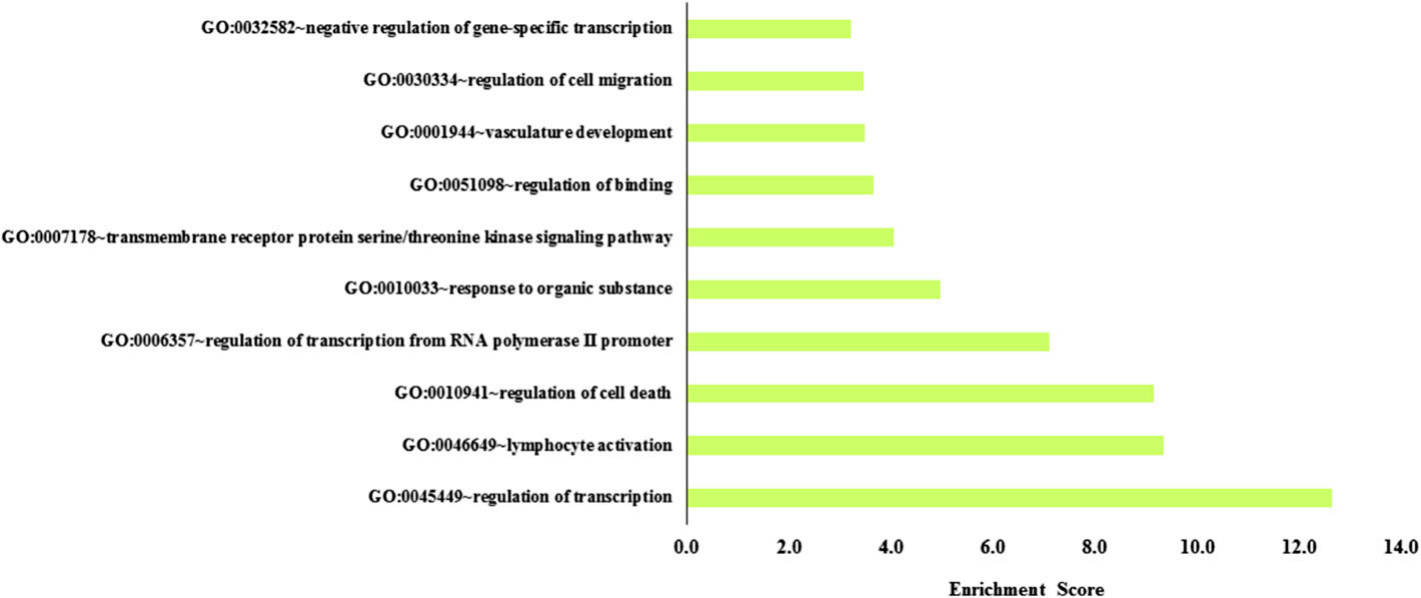
The ten most significant biological processes associated with genes downregulated in relapsed childhood B-ALL

**Fig. 5:**
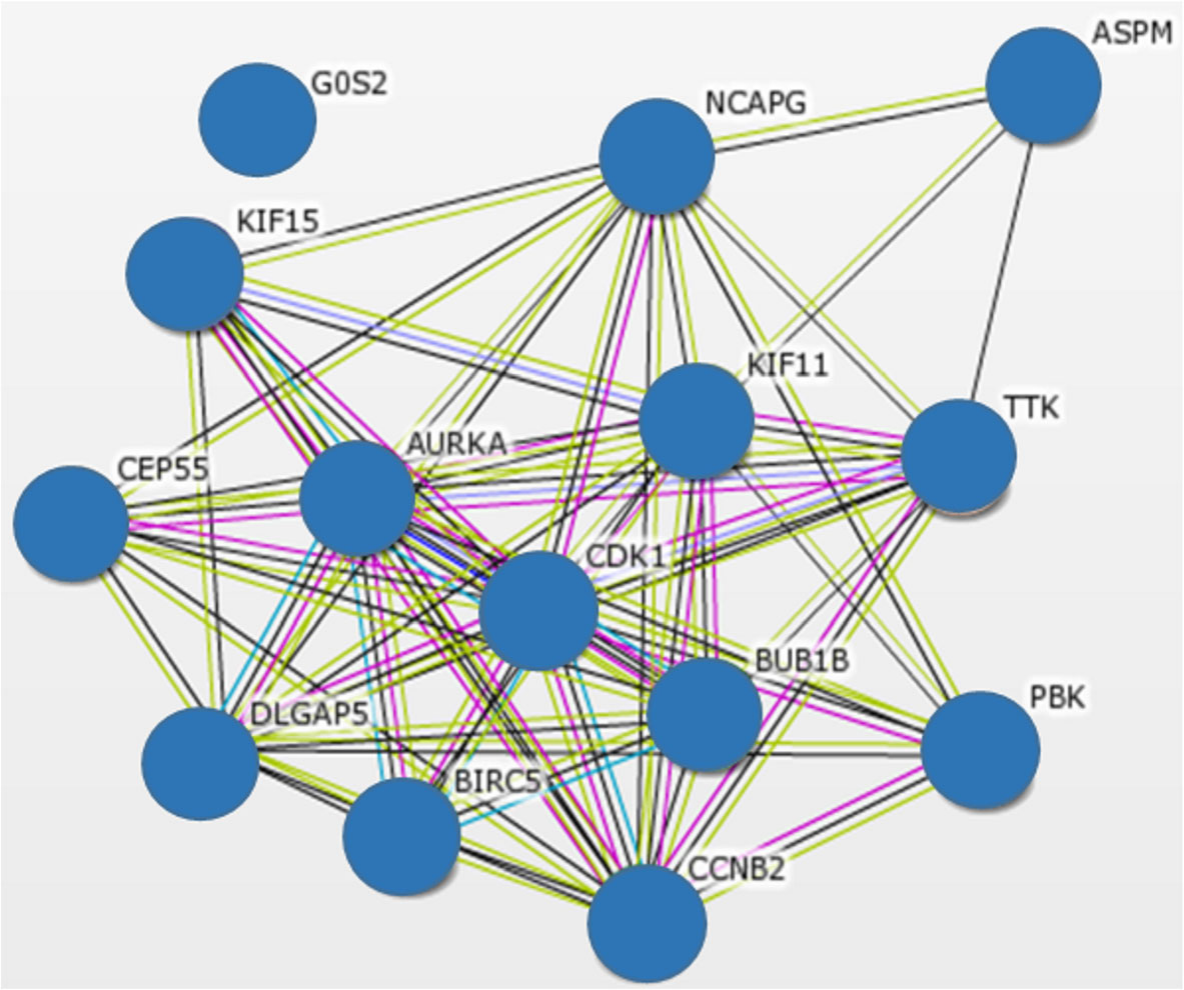
Protein-protein interaction network of cell cycle genes identified in top 100 upregulated probes in relapsed childhood B-ALL

The significantly dysregulated genes were then annotated using DAVID (Additional file 1: Table S3). As depicted in Figs. 3 and 4, based on gene ontology biological process annotation, the 1527 upregulated genes were most enriched in cell cycle processes (enrichment score = 15.3), whilst the 1214 downregulated genes were enriched in transcription regulation (enrichment score = 12.6). Notably, a total of 161 upregulated genes were cell cycle regulators, and many of them (e.g. kinesins, *CDKs*) have been reported to be implicated in leukemia pathogenesis. Of the top 100 significantly upregulated probes, 14 of them (*PBK*, *ASPM*, *AURKA*, *BUB1B*, *BIRC5*, *CDK1*, *CEP55*, *CCNB2*, *DLGAP5*, *KIF11*, *KIF15*, *NCAP5*, *GOS2*, *TTK*) encode for cell cycle regulators and are inter-related via protein-protein interaction network (String network, Fig. 5). Of these candidate genes, *CDK1*, *AURKA*, and survivin (*BIRC5*) are the most attractive candidates, whereby numerous inhibitors under development have entered into either phase I/II clinical trials.

## Discussion

In the past decades, microarray has been used widely to investigate differentially expressed genes and dysregulated pathways underlying cancer pathogenesis. Numerous microarray gene expression studies on pediatric ALL have been performed, with few focused on understanding the biological mechanisms underlying relapsed ALL using matched diagnosis-relapsed samples. Also, each published dataset was relatively small (*n* < 50) and the concordance of these studies is rather low based on the publication findings [7–9] or even with the re-analysis on individual dataset using the limma method (Fig. 1; Additional file 1: Table S1). The discrepancies could be attributed to the small size in each single dataset which is underpowered to identify reliable candidates of interest. Hence, meta-analysis which merges all qualified datasets into a single analysis using a more robust statistical method is preferable to yield more meaningful set of differentially expressed genes and to provide new insights into the biological mechanisms. Meta-analysis on multiple microarray datasets of various diseases has yielded reliable candidates of interest by increasing the statistical power and generalizability [11–13].

Our meta-analysis demonstrated that *S100A8* was the top gene upregulated in relapsed ALL as compared to matched diagnosis. *S100A8* is a member of the S100 multigene family of cytoplasmic EF-hand Ca2 + -binding proteins [23] and was found overexpressed in various cancer types, and is involved in regulating cell proliferation, metastasis and apoptosis [23–27]. In hematological cancers, *S100A8* has been reported to be overexpressed in childhood AML and associated with a worse prognosis [28, 29]. It may be involved in mediating chemoresistance by upregulating autophagy in leukemia cells through promoting the formation of BECN1-PI3KC3 complex [30]. Also, *S100A8* was found overexpressed in the more aggressive ALL subtype, infant B-ALL, as compared to non-infant B-ALL [31], and mediated prednisolone-resistant in MLL-rearranged infant ALL [32]. Preclinical study has demonstrated S100A8 promoted cell growth of murine B-cell leukemia (BJAB) and human T-cell leukemia (Jurkat) lines [33]. Numerous studies have shown inhibition of S100A8 as a viable treatment strategy for cancers, including leukemia [28, 34–37]. For instance, inhibition of S100A8 has shown increased drug sensitivity and apoptosis of leukemic cells [28]. Given that S100A8 acts as an upstream target of EGFR signaling [38], anti-EGFR therapies, including midostaurin, enzastaurin and gefitinib has been proposed as potential therapy for kidney cancer cells which overexpressed S100A8 [35]. Moreover, increased expression of S100A8 mediated the activation of MAPK and NF-κB pathways, and treatment with p38 MAPK inhibitor SB203580 and the NF-κB inhibitor Bay 11-7082 effectively abolished migration and invasion of cancer cells [39]. Other than conferring selective sensitivity to drugs which target mediators of S100A8, the knockdown of S100A8 expression with siRNA or shRNA also showed reduced invasinesss and migration of cancer cells [28, 34, 36, 37]. Taken together, S100A8 is an ideal target for relapsed ALL therapy, and warrants further investigation.

*MPO* appeared as the second top ranked upregulated genes, with a fold change > 2. MPO has been long considered as the hallmark marker for AML cells by the French–American–British and WHO classifications, and has been used clinically to distinguish between AML and ALL. However, several studies reported *MPO* also being expressed in B-ALL cells, and associated with poorer prognosis [40–43]. For instance, infant B-ALL, a subtype which associated with poorer prognosis was shown to have overexpressed MPO, with an incidence rate of 40–60% [42, 44]. Also, B-ALL patients who presented with MPO-positive showed higher incidence of relapse [45], and reduced long-term survival [46]. Our data therefore suggested that MPO may serve as strong indicator for relapse in B-ALL patients. Moreover, silencing of MPO has been shown to effectively induce apoptosis in ovarian cancer cell lines by increasing caspase-3 activity [47]. Inhibition of MPO-overexpressed cells is therefore of clinical interest.

To date, development of cell cycle inhibitors for cancer therapy is actively ongoing. The most attractive inhibitors are those that target cell cyclin dependent kinases (e.g. *CDK1*) and aurora kinases (e.g. *AURKA*, *AURKB*), which are abundantly expressed in various cancer types. Our meta-analysis and several earlier studies have demonstrated that overexpression of cell cycle proteins was prominent and was among the key genetic changes underpinning progression of relapsed childhood B-ALL [7–9]. From the top 100 upregulated genes list, 14 of them are cell cycle regulators and are found to be interactive with each other (Fig. 5). Of those candidates, *CDK1* appeared as a key target. To date, numerous CDK inhibitors have entered into clinical trials (https://clinicaltrials.gov), and have shown promising clinical response in leukemia patients. For instance, AML patients treated with a combination of flavopiridol and two chemotherapeutic agents, cytarabine and mitoxantrone, showed a complete remission rate of 75% [48], as compared to 40–50% with regimens using only conventional chemotherapy [49, 50]. Also, Dinaciclib, a novel inhibitor of CDKs 1, 2, 5, and 9, has been shown to be effective in CLL patients and induced lesser myelosuppression [51]. Recently, the approval by FDA on the use of a CDK inhibitor, palbociclib, in combination with letrozole to treat advanced estrogen positive, HER2 negative breast cancer has strengthen the usefulness of CDK inhibitors as new class of anti-cancer therapies [52]. In pediatric ALL, incorporation of CDK inhibitors into standard treatment regimens is yet to be investigated, and it is believed that clinical trials of CDK inhibitors on relapsed childhood B-ALL may be justifiable options to improve patients’ survival rate.

Another candidate of cell cycle regulators, *AURKA*, was also found in the top 100 upregulated genes list in our meta-analysis. *AURKA* is one of the three aurora kinases (*AURKA*, *AURKB*, and *AURKC*) which play essential roles in cell proliferation, regulating cell cycle transit from G2, formation of the mitotic spindle, centrosome maturation and separation, and cytokinesis [53–55]. Overexpression of *AURKA* has been documented in solid tumors and hematological cancers [56–60]. Higher levels of *AURKA* expression were correlated with higher tumor grade, and poorer prognosis [61–64]. Furthermore, overexpression of *AURKA* mediated resistance to gefitinib, taxol and cisplatin in cancer cells [65–67]. Inhibition of *AURKA* has been shown to increase cisplatin-induced apoptosis [66]. It is noteworthy that more than 30 AURKA inhibitors have been tested in clinical studies [68]. For relapsed and refractory AML patients, an early phase I/II clinical trial on AURKA inhibitor, MLN8237, has shown 13% complete response rate, 11% partial response rate, and 49% stable disease [69]. Given that the levels of *AURKA* expression was elevated in relapsed pediatric B-ALL, it would be worthwhile to investigate the efficacy of AURKA inhibitor in this group of patients.

Earlier studies have identified survivin overexpression as a strong risk factor for relapse in childhood B-ALL [70]. Independent microarray studies using other analysis pipelines have reported survivin as a key gene upregulated in relapsed ALL [7, 8]. Our analysis has strengthened the fact that targeting survivin is a promising therapeutic strategy, and warrants further investigation. Survivin is part of the AuroraB-survivin-INCENP-Borealin/Dasra B complex, an essential component for cell-cycle progression and cytokinesis [71]. It plays an important role in regulating cell proliferation and apoptosis suppression. Survivin was also found to be overexpressed in adult AML and T-cell leukemia [72, 73] as well as childhood AML [74–76], and associated with poorer survival outcome. Upregulation of survivin is mediated by multiple signaling pathways and by the tumor microenvironment including PI3K, MAPK, STAT3, Wnt/-catenin, hypoxia, angiogenesis, and NF-kβ signaling pathways [53, 76–80], hence may serve as an important target for leukemia therapy. Survivin also mediates resistance to chemotherapeutic agents, including vincristine, cisplatin, and tamoxifen in tumor cells [81–83]. Down-regulation of survivin via antisense oligonucleotides was shown to enhance sensitivity of various cancer cell types to cytotoxic agents such as TRAIL [84], cisplatin [85], taxol [86], imatinib [87], as well as to cytotoxicity induced by radiation therapy [88]. To date, several clinical trials on survivin employing different approaches including antisense oligonucleotides, small molecule inhibitors and immunotherapy are in progress ([89–92], http://www.clinicaltrials.gov), and is offered as an treatment option for terminally ill relapsed B-ALL patients within in the context of clinical trial.

Taken together, our meta-analysis on paired diagnosis-relapsed B-ALL has strengthened the evidence for the roles of cell cycle dysregulation as the key component of genetic alterations underpinning disease progression, and can be considered as the promising pathway for new therapeutic intervention. The efficacy of targeted cell cycle therapies to treat relapsed pediatric B-ALL patients shall be further evaluated in the context of clinical trials.

## Conclusion

In summary, our analysis identified *S100A8* as the top most promising biomarker and therapeutic candidate for relapsed childhood B-ALL. Dysregulation of the cell cycle is the key genetic event implicated in relapsed ALL, and an in-depth investigation of the efficacy of cell cycle inhibitors (e.g. CDK inhibitors, and aurora kinases inhibitors) in eliminating relapsed leukemic cells is warranted to improve patients’ survival rate.

## Additional file

Additional file 1: Table S1. List of significantly differentially expressed probes identified in GSE3910, GSE18497, and GSE28460 analyzed by limma approach. **Table S2.** List of significantly differentially expressed probes identified in the meta-analysis of three microarray datasets (GSE3910, GSE18497, GSE28460) using RankProd approach. **Table S3.** Gene set enrichment analysis for the significant upregulated and downregulated genes analzyed by DAVID. (XLSX 248 kb)

## Abbreviations

ALL: Acute lymphoblastic leukemia
AURK: Aurora kinase
CDK: Cyclin dependent kinase
FC: Fold change
logFC: Log2 fold change
pfp: Probability of false prediction
RMA: Robust Multichip Averaging
